# Investigation of the Viromes of Solanaceous Weeds in Hungary Using High-Throughput Sequencing Adds New Insights to Their Hidden Complexity

**DOI:** 10.3390/v18040474

**Published:** 2026-04-17

**Authors:** Burim Ismajli, Zsuzsanna N. Galbács, Lilla Dorottya Péri, György Pasztor, András Péter Takács, Éva Várallyay

**Affiliations:** 1 Genomics Research Group, Department of Plant Pathology, Institute of Plant Protection, Hungarian University of Agriculture and Life Sciences, 2100 Godollo, Hungary; bismajli81@gmail.com (B.I.); nagyne.galbacs.zsuzsanna@uni-mate.hu (Z.N.G.); perililladorottya@gmail.com (L.D.P.); 2 Biological Science Program, Doctoral School of Natural Sciences, Hungarian University of Agriculture and Life Sciences, 2100 Godollo, Hungary; 3 Department of Plant Protection, Institute of Plant Protection, Hungarian University of Agriculture and Life Sciences, 8360 Keszthely, Hungary; pasztor.gyorgy@uni-mate.hu (G.P.); takacs.andras.peter@uni-mate.hu (A.P.T.)

**Keywords:** virome, solanaceous, weed, high-throughput sequencing, pararetrovirus, *Solanum nigrum*, *Solanum dulcamara*, *Datura stramonium*

## Abstract

**Highlights:**

- A survey of randomly collected weeds: namely *Solanum nigrum*, *Datura stramonium* and *Solanum dulcamara* in two consecutive years has been carried out using HTS and validated by and independent method.
- The presence of BBWV1, CMV and PVM in *S. nigrum* and PVM in *S. dulcamara* has been revealed supporting their infection on these hosts.
- *D. stramonium* has been described as a new host of TuYV.
- A tobamovirus, most probably the SDYFV was found in *S. dulcamara* and *D. stramonium*, and evidence was provided that in contrast to the current knowledge, it is a distinct species from Obuda pepper virus (ObPV).
- The presence of a PVH-like and an OxruMV1-like virus has been revealed, which could be a new species, but if not, they are still the first description of these types of viruses from Europe and from *S. dulcamara*.
- The results suggest that tobacco vein clearing virus (TVCV) can be integrated into the genome of *S. nigrum* in several copies, and can also be integrated into the genome of *D. stramonium*, or if not, it can infect this host

**Abstract:**

Weed control of solanaceous weeds growing with solanaceous crops is a constant challenge. Infected by viruses, they can also act as virus reservoirs, complicating this problem further. Viromes of annual *Solanum nigrum*, *Datura stramonium*, and *Solanum dulcamara*, a perennial climbing shrub, were investigated using RNA sequencing and validated using RT-PCR, revealing infection with nine viruses. Broad bean wilt virus 1 (BBWV1), cucumber mosaic virus (CMV), and potato virus M (PVM) were found to infect *S. nigrum*. Investigating only 46 plants revealed infection with *Solanum dulcamara* yellow fleck virus (SDYFV) not only in *S. dulcamara* but in a new host, *D. stramonium*, which also represents a new host of turnip yellows virus (TuYV). We described the first presence of a potato virus H (PVH)-like, and Oxybasis rubra mitovirus 1 (OxruMV1)-like virus in Europe, in *S. dulcamara* as a new host. Our results highlight the unexpected complexity of the viromes of solanaceous weeds, which should be considered during reliable and efficient plant protection strategies, in order to alleviate the virus reservoir role of the weeds.

## 1. Introduction

Crop production faces numerous biotic challenges that impair plant growth and development, including competition for light, nutrients, and water. The presence of weeds, as competitors, poses a major problem to solve, which is why herbicides are used in large quantities. Weeds are not only competitors but also serve as reservoirs of plant pathogens. Viral infections can alter and affect plant physiology, which can lead to the development of different symptoms such as chlorosis, mosaic patterning, stunting, and deformities, and cause yield losses and economic damage. Weeds can also serve as food and shelter for insects, which can vector the present viruses, accelerating their spread. Climate change and globalisation can reshape the presence and spread of weeds and viral vectors, and consequently the viromes of the crop lands [1].

Solanaceous weeds, growing in potato, tomato and pepper fields, compete with the crop, and as they belong to the same family as the crop itself, herbicide control against them is extremely difficult. They can host the same range of viruses as the crop, and threaten its yield, if they are virus-infected and act as a virus reservoir (recently reviewed [2].

*Solanum nigrum*, black nightshade, is an annual weed that grows in different habitats, native to Eurasia. It is a very aggressive competitor and can produce a large number of seeds, which can easily germinate in the next year. It is highly resistant to different abiotic stresses and can also develop resistance to herbicides [3]. It can be infected by several viruses and is suspected to have a virus reservoir role within and at the edges of the crop fields. This role has been investigated in Tunisia [4] and India [5] with traditional techniques, and in France, using HTS [6].

*Datura stramonium*, jimsonweed, is an annual, invasive plant with high vigour and massive biomass, which is also poisonous, as it produces tropane alkaloids. Investigation of its adaptation to elevated CO_2_ and temperature suggests that it will invade new habitats in the future [7].

In contrast to the above species, *Solanum dulcamara*, bittersweet nightshade, is a perennial climbing shrub. It prefers wet, marshy areas, but can adapt to drier locations. It is native to Europe and Asia but has become a widespread and invasive weed in North America.

Viromes of weeds have been investigated for a long time. As their infection is usually latent, the first reports about viruses of weeds in Hungary have been described in rare cases, when they caused symptoms [8], or when they were artificially infected. *S. nigrum* was found to be a new experimental host of Melandrium yellow fleck (MYFV) and sowbane mosaic virus (SoMV) [9], while *D. stramonium* was proven to be the host of cucumber mosaic virus (CMV) and Henbane mosaic virus (HeMV) [10]. Moreover, the effect of virus infection on germination and plant physiology was characterised in tobacco mosaic virus (TMV), Pepino mosaic virus (PeMV) and Obuda pepper virus (ObPV) infected *S. nigrum* [11,12]. Susceptibility to a particular virus does not mean that the weed’s natural population is highly infected with it. As follow-up research, solanaceous weeds in their natural habitats in Hungary were tested for the presence of viruses using serological methods. Investigating viromes of *S. nigrum* revealed its infection with several viruses, including potato virus A (PVA), potato virus X (PVX), potato virus Y (PVY), TMV and tomato mosaic virus (ToMV) [11], while *D. stramonium* was infected with HeMV [12].

Further development of the virus diagnostic techniques offered unbiased methods for virus detection, like macroarray, though the number of tested viruses with it was still limited. Using a potato virus detecting macroarray in *S. dulcamara* samples revealed infection with potato virus M (PVM) [13].

The revolution in sequencing techniques and the emergence of high-throughput sequencing (HTS) opened new possibilities to discover the viromes of plants [14]. HTS revealed the presence of a previously unknown virus species in a large number, especially when it was used to investigate the virome of weeds, which includes sampling natural habitats [14,15,16] (recently reviewed) [2]. The virome of solanaceous plants has been reinvestigated, with new insights and undescribed viral infections being revealed [17].

During our study, several viruses were identified (Table 1).

*BBWV-1* has a wide host range, including important crops and ornamental plants, and has been reported from all over the world. Despite this high prevalence, the number of its nucleotide sequences available in the GenBank is limited [18]. CMV has an extraordinarily wide host range, infecting over 1200 plant species [46]. TuYV is one of the most important viruses infecting oilseed rape, causing severe yield loss worldwide, and is present in Hungary [26]. It has a very wide host range, including several different weed species, highlighting that solanaceous weeds may also contribute to the maintenance and spread of the virus within agricultural ecosystems. Together with Mirafiori lettuce big-vein virus (MiLBVV), LBVaV is involved in lettuce big-vein disease (LBVD), which is characterised by reduced growth, mosaic discolouration, chlorotic vein banding, and leaf deformations on lettuce, leading to up to 70% yield loss [32]. ObPV is a virus that can break the N-gene resistance in tobacco, and this feature could have been connected to a single nucleotide change in the 126 kDa replicase gene [47,48]. It could infect *S. nigrum* artificially, and it was found that the infection results in biomass decrease and could contribute to the reduction in the weed population [35]. Although in most of the cases, it does not induce symptoms, the presence of PVM infection can lead to yield loss, and this is why its presence should be avoided in the potato propagation material [49]. It can also infect the tomato [50,51,52] and also peppino [48]. Surveying potato fields in Transylvania, we have found its presence in potato at a location with low weed control, occupied with a high density of aphids, while it was missing from the potatoes, when high intensity weed control could efficiently regulate the size of the aphid population [53]. PVH has not been reported from outside of Asia. This is why it is stated to be non-EU in the last EFSA report about pest categorisation of potato viruses and viroids [54].

Viruses, which can replicate in the mitochondria (mitoviruses), are widespread among fungi and have been recently discovered in plants [55]. Their positive-sense RNA genome encodes only one protein, the viral RdRP. As they replicate in the mitochondria and are transmitted to daughter cells during cell division, they do not need either coat or movement protein for their proper function [43]. The first mitoviruses have been described as pure sequences, using in silico methods, and during this study, several mitoviruses, including OxruMV1, were identified [43]. The existence of a *bona fide* plant mitovirus and its replication in the host mitochondria were demonstrated later, in the case of Chenopodium quinoa mitovirus 1 [55]. An unbiased survey of metagenomes from soil samples identified viruses in an unexpected number, with several putatively plant-infecting viruses among them [56]; however, the role of these viruses in plant life and health has just started to be investigated.

TVCV can be integrated exclusively present in the genome of Solanaceae hosts: *Nicotiana* species, *S. lycopersicum*, and *S. tuberosum* [44], was described from a *N. edwardsonii* showing vein-clearing symptoms [45]. Knowledge about the viromes of *S. nigrum*, *D. stramonium* and *S. dulcamara* has been widened in the past decade, and sequences of the infecting virus variants are now available in the NCBI Genbank in a growing number; however, molecular studies are still limited and have not yet been carried out in Hungary.

In this study, we surveyed fields in the close vicinity of Keszthely. We sampled *S. nigrum*, *D. stramonium* in crop fields and *S. dulcamara* at a natural habitat in two consecutive years, to investigate their viromes using unbiased HTS. Our results revealed new descriptions of the viromes of these solanaceous plants.

## 2. Materials and Methods

### 2.1. Plant Material and Nucleic Acid Extraction

During the summer of 2022 and 2023, symptomatic solanaceous weeds were surveyed in Keszthely, Hungary, to characterise their viromes. In both years, sampling was conducted at two locations. In 2022, Field I, Field II, and Field III in 2023 were agricultural fields, while Field IV, sampled in 2023, was situated at the lakeside of the Balaton. In 2022, we sampled asymptomatic plants: five *S. nigrum* plants at Field I and Field II and five *D. stramonium* plants at Field I (Table A1). In 2023, we searched for symptomatic plants and collected samples from ten *S. nigrum*, ten *D. stramonium*, and three *Brassica napus* at Field III. At the lakeside, we sampled eight *S. dulcamara* exhibiting virus-like symptoms. Total nucleic acid was extracted from the frozen leaves, from each sample individually, using a phenol-chloroform method [57].

### 2.2. Sample Preparation for RNAseq and sRNA Sequencing Library Preparation

Total nucleic acids from the same species originating from the same location were pooled. For the pooling strategy, see Table A1. The pools were further mixed, and finally, two pools representing the sampling year were prepared. For RNA-Seq, the nucleic acids of the pools were DNase-treated using Thermo Scientific DNase I, RNase-free (Thermo Fisher Scientific, Waltham, MA, USA), following the manufacturer’s recommendation. The DNase-treated sample, containing high-quality pure RNA, was named SOLKES1 and SOLKES2 and sent for ribodepleted RNA sequencing, which was ordered as 150 bp paired-end ncRNA sequencing on the Illumina platform from NOVOGEN as a service. Fatsq files of the sequenced reads were deposited into NCBI SRA (BioProject ID PRJNA1401399, BioSample ID SAMN54561009, SAMN54561010). For sRNA, sRNA fractions from the pools were separated on a polyacrylamide gel and purified. The sRNA sequencing library, named KSOL, was prepared according to our updated in-house protocol [58], based on the TruSeq Small RNA Library Preparation Kit (Illumina, San Diego, CA, USA). The sRNA library (prepared only from the samples collected in 2022) was sequenced using single-indexed 50 bp single-end reads on a HiScanSQ platform (UD-Genomed, Debrecen, Hungary). The resulting fastq file has been deposited into the NCBI SRA database (BioProject ID PRJNA1401399, BioSample ID SAMN54561009).

### 2.3. Bioinformatic Analysis of the HTS Results

FASTQ files from the HTS were analysed using CLC Genomic Workbench (version 20.0.4., Qiagen, Hilden, Germany). For RNASeq, the reads were trimmed and quality-checked using the Trim reads and QC Report tool. Paired reads were then assembled de novo into contigs (see Table S2 for initial statistics). BLAST (version 2.16) analysis of the contigs against reference genomes of the known plant-infecting viruses (downloaded from NCBI GenBank, 31 July 2023) identified the viruses present in the sampled plants. Reads were also directly mapped to the reference genomes of viruses for which contigs with zero E-value were obtained. Consensus sequences generated from these mappings were used to calculate the coverage of the viral genomes. For sRNA, after trimming and quality control, non-redundant reads were assembled into contigs using the CLC de novo assembler with default parameters (word size 20, bubble size 50, minimum contig length 35 nt) (Table S1). Contigs were annotated using BLASTN (default settings: thread 1, word size 11, match 2, mismatch 3, gap cost existence 5, extension 2) against the NCBI Plant Viral Reference genomes (downloaded 31 July 2023) and a collection of 165 virus sequences from rural samples identified in Slovenia [20]. For viruses represented by at least one contig, reads were mapped to the reference genome, allowing one mismatch, and counts were obtained with and without redundancy. Normalised redundant reads (reads per million, RPM) were calculated based on the mapped reads and total sequenced reads. Consensus sequences were generated from the mappings and used to calculate genome coverage (%) (Table S2).

### 2.4. Validation of the HTS by RT-PCR

To validate the results of the bioinformatic analysis, RT-PCR was conducted. RNA, which was used for HTS and RNA of all pools and all individual samples were used as templates for cDNA synthesis. cDNA synthesis was performed using the RevertAid First Strand cDNA Synthesis Kit (Thermo Fisher Scientific, Waltham, MA, USA) with random primers, following the manufacturer’s instructions.

The quality of the cDNA was confirmed by PCR with actin-specific primers. Virus-specific primers were utilised to amplify various genomic regions of the virus of interest. The primers used for the amplification were from published papers or designed based on the contig sequences (Table S3). RT-PCR was performed using Q5 Hot Start High-Fidelity DNA Polymerase (New England Biolabs, Ipswich, MA, USA) and Phire Hot Start II DNA Polymerase (Thermo Fisher Scientific, Waltham, MA, USA), following the manufacturer’s protocols. The optimal annealing temperature of the primers was determined experimentally. Virus-specific PCR products were purified using the NucleoSpin Gel and PCR Clean-up Kit (Macherey and Nagel, Dueren, Germany), cloned into GeneJET vectors (Thermo Fisher Scientific, Waltham, MA, USA), and Sanger sequenced, which was ordered from Eurofins BIOMI Kft (Godollo, Hungary), as a service. Sequences were deposited into GenBank (Accession numbers: PZ011567-PZ011584, and PZ051109-16). In case of the same sequence originating from different hosts, only one sequence variant/virus has been deposited. A detailed list of the sequenced which were deposited into the NCBI GenBank can be found in Table S4. Coverage of viral genomes by sequenced sRNAs was determined for all viruses identified by RNA-seq, regardless of initial bioinformatic results. The size distribution of virus-mapped reads was assessed using the QC report in CLC Genomic Workbench.

### 2.5. Phylogenetic Analysis of the Detected Viral Strains

To compare and phylogenetically analyse the virus variants detected in the samples, multiple sequence alignments were generated in Geneious Prime (version 2024.0.7. using the MUSCLE algorithm. Evolutionary relationships were inferred using the Tamura Nei model and the Neighbour-Joining method. Phylogenetic trees were constructed using the optimal model for each alignment and were tested with 1000 bootstrap replicates to estimate the significance of the nodes. Branch lengths represent the number of substitutions per site.

## 3. Results

### 3.1. HTS of the Solanaceous Weeds Indicated the Presence of Several Different Viruses

SOLKES1 contained 16,253,182 raw reads, and 15,972,388 high-quality reads remained after trimming and quality control (Table S1). *De novo* assembly of these reads resulted in 132,523 contigs. In the SOKES2, out of the 23,061,440 raw reads, 22,913,754 high-quality trimmed reads remained after trimming and quality control, which could be de novo assembled into 71,700 contigs (Table S1). BLAST analysis of the contigs of SOLKES1 and SOLKES2 identified the presence of four and seven viruses with significant homology (contig hits with only E-value = 0 were considered), respectively (Table S2). In 2022, we got hits to BBWV1, CMV, TuYV, and TVCV; in 2023, to LBVaV, ObPV, PVM, PVH, OxruMV1, PotLV, and TVCV.

To check the viral origin and validity of the contigs, we BLAST-ed them individually, which revealed that they are valid, except for hits to PotLV. The two contigs, which were originally BLAST-ed to the PotLV genome, were very short: 1651 nt and 1353 nt long and could also be annotated as PVM, suggesting that they were mistakenly annotated as PotLV.

Mapping of viral reads to the reference genomes showed high coverage (>70%) of the viral genome, except for ObPV, PotLV, and TVCV (2023), which had coverage of 33%, 31%, and 45%, respectively.

**Figure 1:**
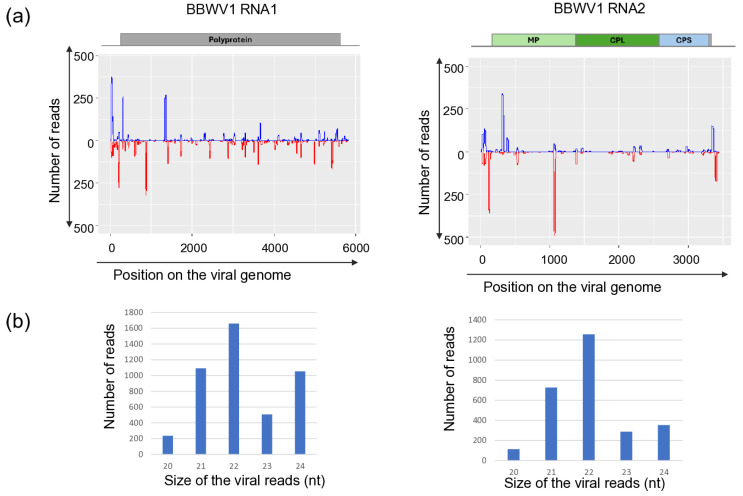
Investigation of the mapped viral reads in the case of BBWV1 RNA1 and RNA2. (**a**) The coverage of the viral genome by sRNAs (blue indicates sRNAs in sense, while red indicates antisense orientation, (**b**) the size distribution of the viral reads.

**Figure 2:**
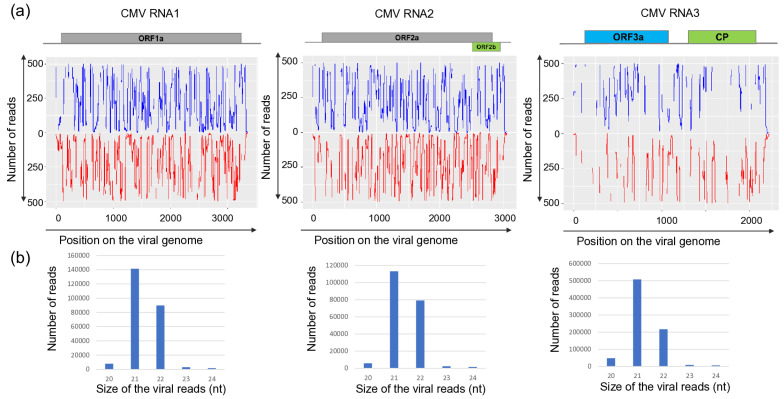
Investigation of the mapped viral reads in the case of CMV RNA1, RNA2 and RNA3. (**a**) The coverage of the viral genome by sRNAs (blue indicates sRNAs in sense, while red indicates antisense orientation, (**b**) the size distribution of the viral reads.

**Figure 3:**
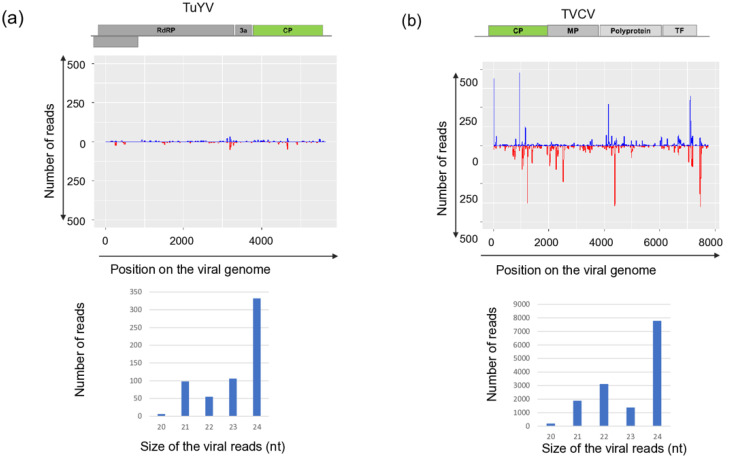
Investigation of the mapped viral reads in the case of (**a**) TuYV and (**b**) TVCV. **Upper** panel: The coverage of the viral genome by sRNAs (blue indicates sRNAs in sense, while red indicates antisense orientation, **lower** panel the size distribution of the viral reads.

Sequencing KSOL resulted in 21,955,551 reads, of which 21,573,415 high-quality redundant reads remained after trimming, representing 3,178,957 non-redundant reads (Table S1). The assembled 3177 contigs were BLAST-ed to the reference genomes of the viruses and resulted in the same hits that we got using RNAseq. Mapping of the sRNA reads showed that sRNAs, both in sense and antisense positions, covered each segment of BBWMV1 (Figure 1) and CMV genomes (Figure 2), and the genomes of TuYV and TVCV (Figure 3).

While the size of the sRNAs originating from BBWV1 and CMV was predominantly 22 and 21 nt long, respectively, the sRNAs generated from the TuYV and the DNA genome of TVCV were mostly 24 nt long.

The number of mostly 21 nt long redundant sRNAs mapped to the viral genome was very high in the case of CMV: with 234,654, 190,706, 804,366 for RNA1, RNA2 and RNA3, respectively, indicating the active phase of the viral infection. During this phase, the virus, present in high concentration, induces the antiviral RNA of the host, activating the DCL1 and DCL4 [59]. Products of these DICER enzymes are mostly 21–22 nt long siRNAs, which we found here (Figure 2). The number of redundant sRNAs mapped to BBWV1 was significantly lower, and their size was predominantly 22 nt long, indicating the more persistent nature of the infection and the high activity of DCL2 in this case (Figure 1). A striking contrast to these cases was found for TuYV and TVCV, where a low number of sRNAs mapped to the viral genome was found, and their size was mostly 24 nt long (Figure 3), indicating a persistent infection and the activity of DLC3.

### 3.2. RT-PCR Validation of the RNAseq Confirmed Infections by Nine Viruses

To validate the result of the RNAseq and sRNA HTS, an independent method, RT-PCR, was used. Virus-specific primers were designed based on the contig sequences to increase the possibility of perfect annealing. For the validation, both the RNA of the pools, which were used for the HTS, the pooled RNA of the same species and the RNA of all of the sampled individuals were tested. This way, we could determine the infection status of all of the sampled individuals. Results are discussed below according to the viral hits.

#### 3.2.1. BBWV1

**Figure 4:**
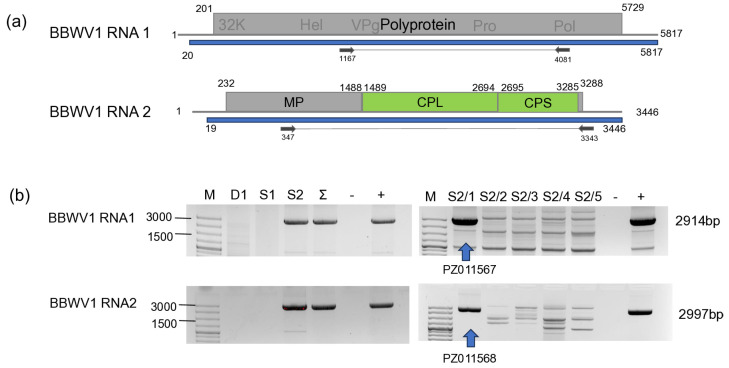
Summarised result of the HTS and its validation for the presence of BBWV1 using RT-PCR. (**a**) Cartoon representation of the BBWV1 genome and the position of the primers used for RT-PCR validation. The dark blue line indicates the region covered by the contig. (**b**) Shows the result of the validation of the presence of BBWV1. M—GeneRuler 100 bp Plus DNA Ladder, D1—indicates the *D. stramonium* pool, S1—*S. nigrum* pool of field I, S2—the *S. nigrum* pool of field II, Σ—denotes the combined pool, which was sequenced, −/+ stand for negative and positive controls, S2/1, 2, 3, 4, and 5 denote individuals of *S. nigrum* from field II. The arrow shows the product whose sequence was deposited into the NCBI GenBank, indicating its accession number.

Validation of BBWV1 by RT-PCR revealed that only *S. nigrum* at Field II, and only one plant, S2/1, was infected with this virus (Figure 4). The PCR product of the BBWV1 RNA1 obtained from the infected sample was directly sequenced, whereas RNA2 was cloned and subsequently Sanger sequenced (PZ011567-68).

**Figure 5:**
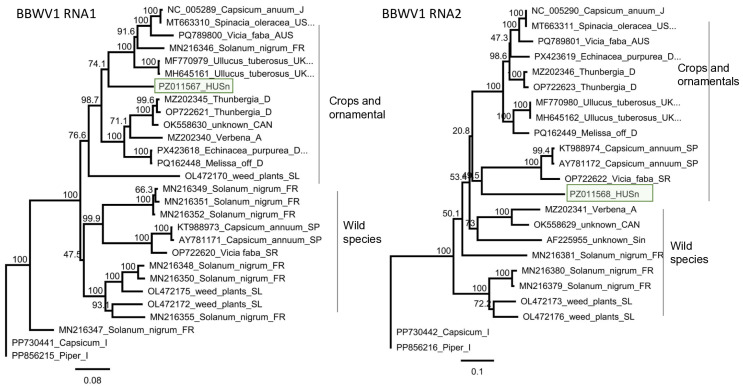
Phylogenetic analysis of the BBWV1 variants: RNA1 and RNA2. The analysis was conducted using Geneious Tree Builder with the Tamura-Nei model, the Neighbour-Joining method, and 1000 bootstrap replicates. The green box highlights the HUSn variant. The sequences are indicated by their GenBank accession numbers, the name of the host plant species, and their geographical origin with an abbreviation of the country of origin: UK—Great Britain, CAN—Canada, FR—France, D—Germany, USA—United States of America, AUS—Australia, A—Austria, J—Japan, SL—Slovenia, I—Italy, SR—Syria, SP—Spain, and Sin—Singapore.

Sequence analysis of the HUSn variant showed that while RNA1 was most similar (85% identity) to the variant which was sequenced in an imported *Ullucus tuberosus* plant in the UK [60], the closest homologue, based on the RNA2 sequence, was the reference strain, which was sequenced from *C. annuum* in Japan [61] (Table S5). The identity of the HUSn compared to the strains available at the GenBank was very low: 81.2–85% and 79.4–85% for RNA1 and RNA2, respectively. Phylogenetic analysis based on RNA1 and RNA2 showed a different clustering of the HUSn strain. RNA1 clustered with the reference strain and strains originating mostly from crops and ornamentals, while RNA2 clustered together with the strains that were sequenced in wild species, including *S. nigrum*, suggesting its possible reassortant origin (Figure 5).

#### 3.2.2. CMV

**Figure 6:**
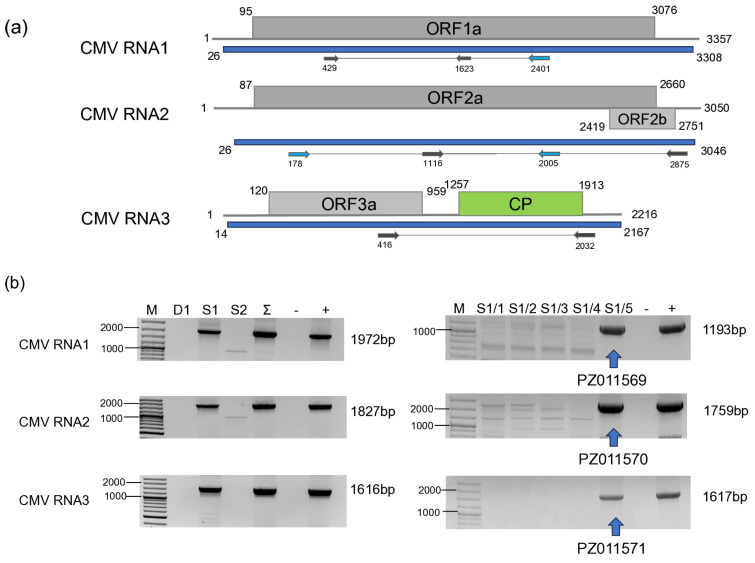
Summarised result of the HTS and its validation for the presence of CMV. (**a**) Schematic representation of the CMV genome, and the position of the primers used for RT-PCR validation. The dark blue line indicates the region covered by the contigs (**b**), the result of the RT-PCR validation of the virus. M—represents the GeneRuler 100 bp Plus DNA Ladder, D1—indicates the *D. stramonium* pool, S1—the *S. nigrum* pool of field I, S2—the *S. nigrum* pool of field II, Σ—denotes the combined pool of D1, S1, and S2, which was sequenced, −/+ stand for negative and positive controls, S1/1, 2, 3, 4 and 5 denotes individuals of *S. nigrum* from Field I. The arrows show the products whose sequence was deposited into the NCBI GenBank, indicating their accession numbers.

CMV was found to infect only one individual of *S. nigrum*, S1/5, in 2022 (Figure 6).

**Figure 7:**
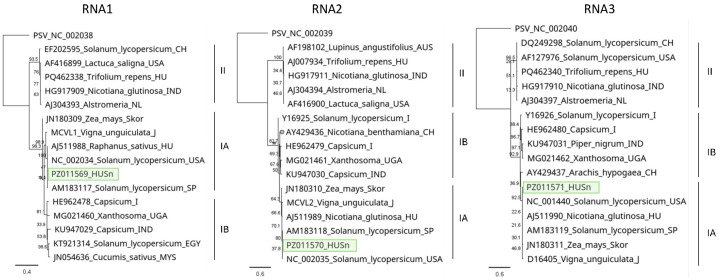
Phylogenetic analysis of the CMV variants:RNA1, RNA2 and RNA3. The analysis was conducted using Geneious Tree Builder with the Tamura-Nei model, the Neighbour-Joining method, and 1000 bootstrap replicates. The green box highlights the HUSn variant. The sequences are indicated by their GenBank accession numbers, the name of the host plant species, and their geographical origin with an abbreviation of the country of origin: UK—Great Britain, CAN—Canada, FR—France, D—Germany, USA—United States of America, AUS—Australia, A—Austria, J—Japan, SL—Slovenia, I—Italy, SR—Syria, SP—Spain, Sin—Singapore, UGA—Uganda, EGY—Egypt, MYS—Malaysia.

Partial RNA 1, RNA2 and RNA3 were cloned, Sanger-sequenced (PZ11569-71) (Table S4), and used for phylogenetic analysis. Sequences of both RNA1, RNA2 and RNA3 clustered uniformly into the IA group (Figure 7), containing the reference Fny, and the virulent strains.

RNA1 of the HUSn strain showed 99% identity with the Fny strains, and RNA2 was almost identical to the RNA2 of the Rs strain found in *Raphanus sativus* in Hungary [62], while the closest CMV sequence to the RNA3 derived from the DSMZ PV-1414 isolate originated from a *Cucurbita pepo* from the USA (OR607780) (Table S6).

#### 3.2.3. TuYV

**Figure 8:**
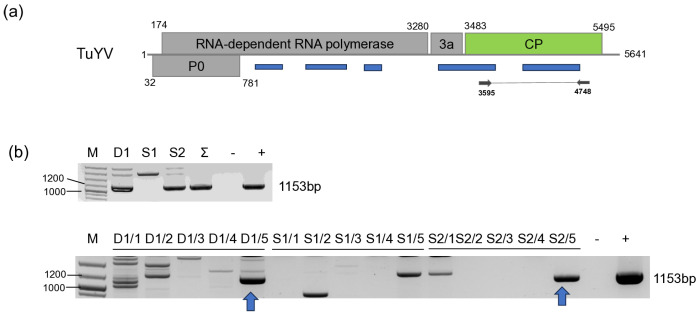
Summarised result of the HTS and its validation for the presence of TuYV. (**a**) Schematic representation of the TuYV genome, and the position of the primers used for RT-PCR validation. The dark blue line indicates the region covered by the contigs. (**b**) The result of the RT-PCR validation of the virus. M—represents the GeneRuler 100 bp Plus DNA Ladder, D1—indicates the *D. stramonium pool*, S1—the *S. nigrum* pool of field I, S2—the *S. nigrum* pool of field II, Σ—denotes the combined pool of D1, S1, and S2, which was sequenced, −/+ stand for negative and positive controls, D1/1–5, S1/1–5 and S2/1–5 indicate the plants sampled in 2022. The arrows show the products whose sequence was deposited into the NCBI GenBank. As the sequence of the two products was the same, they share their GenBank accession number: PZ011584. PCR products appearing in the case of D1/2, S1/5 and S2/1 were aspecific.

We found one *D. stramonium* (D1/5) and one *S. nigrum* (S2/5) plant infected with TuYV (Figure 8).

**Figure 9:**
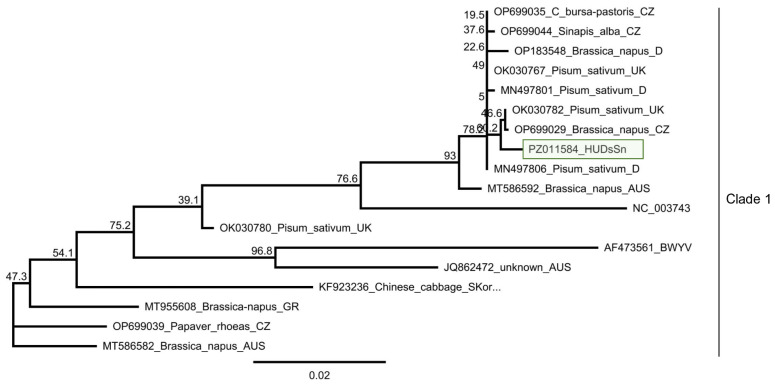
Phylogenetic analysis of the TuYV HUDsS variant. The analysis was conducted using Geneious Tree Builder with the Tamura-Nei model, the Neighbour-Joining method, and 1000 bootstrap replicates. The green box highlights the HUSn variant. The sequences are indicated by their GenBank accession numbers, the name of the host plant species, and their geographical origin with an abbreviation of the country of origin: CH—China, AUS—Australia, Skor—South Korea, J—Japan, D—Germany, GR—Greece, UK—Great Britain, and CZ—Czech Republic.

Sanger sequencing of the cloned PCR products showed that they are identical. This HUDsSn variant was very similar (99% identity) to the NoA9melnikCZ variant, which was sequenced in the Czech Republic in *Brassica napus* [27] and shared more than 99% identity with all variants sequenced in that study, indicating that the TuYV variants present in natural habitats in weed hosts have very similar sequences in Central Europe (Table S7). It clusters to Clade 1 of the virus, where most of the strains sequenced in Europe belong [25] (Figure 9).

#### 3.2.4. LBVaV

**Figure 10:**
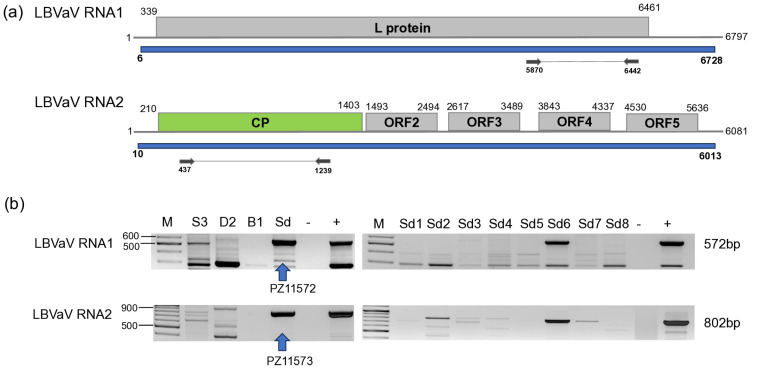
Summarised result of the HTS and its validation for the presence of LBVaV. (**a**) Schematic representation of the LBVaV genome and the positions of the primers used for RT-PCR validation. The dark blue line indicates the region covered by the contigs. (**b**) shows the result of the validation of the presence of LBVaV RNA1 and RNA2 using RT-PCR. M—represents the GeneRuler 100 bp Plus DNA Ladder, S3 indicates the *S. nigrum* pool, D2—*D. stramonium* pool, B1—*B. napus* pool from Field III, Sd—*Solanum dulcamara* pool from Field IV. −/+ stand for negative and positive controls, Sd/1–8 denotes individuals of *S. dulcamara* at Field IV. The arrow shows the products that were sequenced.

LBVaV was found in one *S. dulcamara* individual (Sd6) at Field IV (Figure 10).

**Figure 11:**
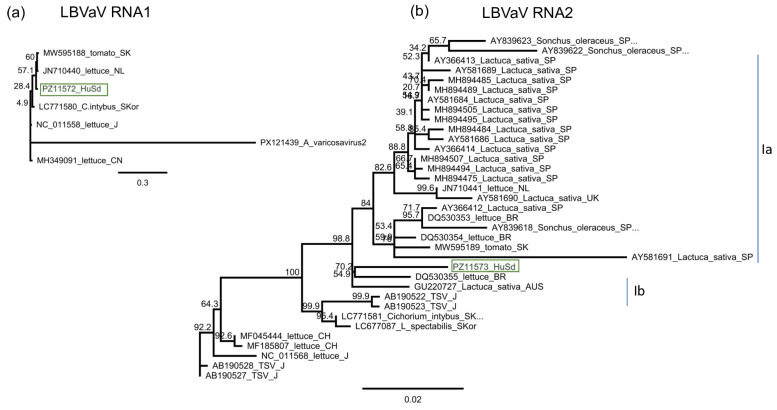
Phylogenetic analysis of the LBVaV (**a**) RNA1 and (**b**) RNA2 HUSn variant. The analysis was conducted using Geneious Tree Builder with the Tamura-Nei model, the Neighbour-Joining method, and 1000 bootstrap replicates. The green box highlights the HUSn variant. The sequences are indicated by their GenBank accession numbers, the name of the host plant species, and their geographical origin with an abbreviation of the country of origin: J—Japan, SK—Slovakia, SP—Spain, CH—China, Skor—South Korea, NL—Netherlands, AUS—Australia, BR—Brazil, and UK—Great Britain.

As only one individual was infected and the sequencing of the PCR products amplifying partial RNA1 and RNA2 was uniform, the sequences of the PCR products were deposited into the NCBI GenBank (PZ11573-4). RNA1 and RNA2 sequences of the HUSd variant were most identical to a variant which was sequenced in the *L. sativa* host in the Netherlands and in Australia, respectively (Tables S4 and S8), but the variability of the LBVaV is low, and the identity of the strains is more than 96%. RNA1 of the HUSD variant clustered with the variant sequenced in tomato in Slovakia, while RNA2 clustered outside Clade 1a, which was suggested to contain the strains present in Europe, while also containing the tomato strain (Figure 11). It instead clustered to Clade 1b, where strains sequenced in lettuce in Brazil and Australia clustered, but separately from the strains belonging to the Asian clade, sequenced in Japan and South Korea.

#### 3.2.5. ObPV-like Tobamovirus

**Figure 12:**
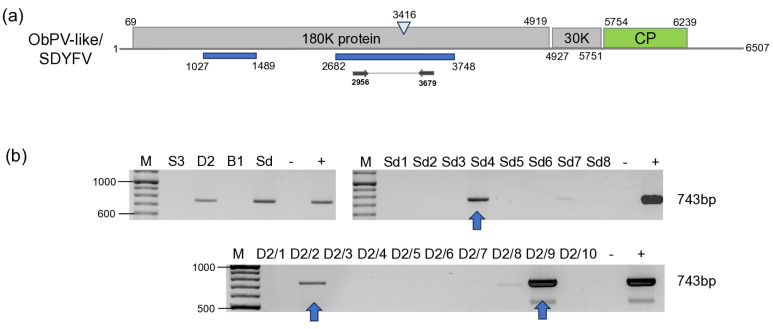
Summarised result of the HTS and its validation for the presence of ObPV-like tobamovirus. (**a**) Schematic representation of the ObPV genome and the positions of the primers used for RT-PCR validation. The dark blue line indicates the region covered by the contigs. (**b**) shows the result of the validation of the presence of ObPV using RT-PCR. M represents the GeneRuler 100 bp Plus DNA Ladder; S3 indicates the *S. nigrum* pool; D2—*D. stramonium* pool; B1—*B. napus* pool from Field III; Sd—*S. dulcamara* pool from Field IV. −/+ stands for negative and positive controls; Sd 1–8 denotes individuals of *S. dulcamara* at Field IV, while D2/1–10 denotes individuals of *D. stramonium* at Field III. The arrows show the PCR products, which were cloned and sequenced.

BLAST of the contigs indicated the presence of ObPV, a tobamovirus, which was represented by two contigs. The contigs aligned to the RdRp coding region, but the 47 reads covered only 33% of the ObPV genome. RT-PCR using the primers, which were designed based on the contig sequences, showed that two *D. stramonium* (D2/2 and D2/9) and one *S. dulcamara* (Sd4) were infected with this virus (Figure 12).

**Figure 13:**
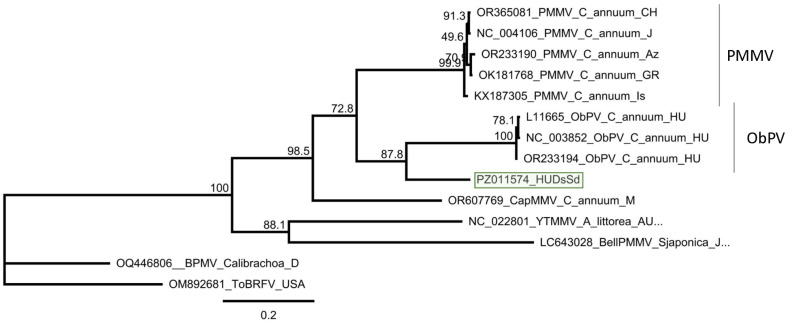
Phylogenetic analysis of the ObPV-like HUDsSd variant (PZ011574). The analysis was conducted using Geneious Tree Builder with the Tamura-Nei model, the Neighbour-Joining method, and 1000 bootstrap replicates. The green box highlights the HUSd variant. The sequences are indicated by their GenBank accession numbers, the name of the host plant species, and their geographical origin with an abbreviation of the country of origin: J—Japan, CH—China, AUS—Australia, HU—Hungary, M—Morocco, Az—Azerbaijan, USA—United States of America, GR—Greece, and Is—Israel.

Cloning and sequencing the amplified product showed that the variants present in the three individuals are the same (at least within the sequenced region) (PZ011574). In the GenBank, there are three full genomes of ObPV, but although they were sequenced at different laboratories, all of them originate from Hungary (L11665, NC_003852 and OR233194). The HUDsSd variant showed the highest identity to OR233194 (Sequenced at DSMZ), but the identity was only (585/743) 79% on the nucleotide (Table S9) and (226/247) 91% on the amino acid level. The amplified part showed was just a bit lower, 77% (572/743) identity to a variant of PMMV (OK181768—sequenced from a pepper in Greece), which was 88% on the amino acid level. Sequence identity of the HUDsSd is lower than the species demarcation criterion in the genus Tobamovirus, which is 90% at the nucleotide level, suggesting to us that we did not find ObPV, but rather a new, ObPV-like tobamovirus in the sampled plants. The sequence of this ObPV-like virus clustered with ObPV, suggesting its close relationship with this virus (Figure 13).

#### 3.2.6. PVM

**Figure 14:**
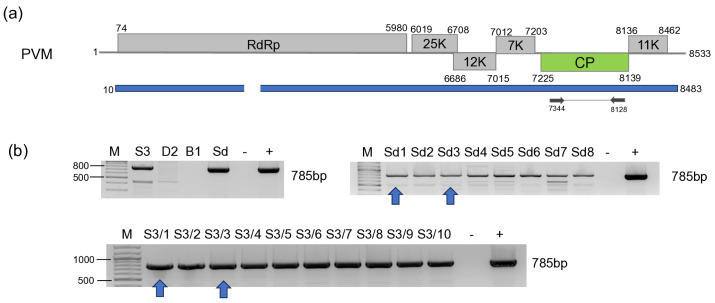
Summarised result of the HTS and its validation for the presence of PVM. (**a**) Schematic representation of the PVM genome and the positions of the primers used for RT-PCR validation. The dark blue line indicates the region covered by the contigs; (**b**) shows the result of the validation of the presence of PVM using RT-PCR. M represents the GeneRuler 100 bp Plus DNA Ladder; S3 indicates the *S. nigrum* pool of Field III; D2—*D. stramonium* pool of Field II; B1—*B. napus* pool of Field II; Sd—*S. dulcamara* pool; −/+ stands for negative and positive controls; Sd 1–8 denotes individuals of *S. dulcamara* at Field IV, while S3/1–10 denotes individuals of *S. nigrum* at Field III. The arrows show the PCR products, which were cloned and sequenced.

PVM-derived reads covered 82% of the viral genome. RT-PCR validation showed that all of the individuals of *S. nigrum* at Field III and *S. dulcamara* at Field IV sampled in 2023 were infected with this virus (Figure 14), while no infection with this virus was detected in the previous year.

**Figure 15:**
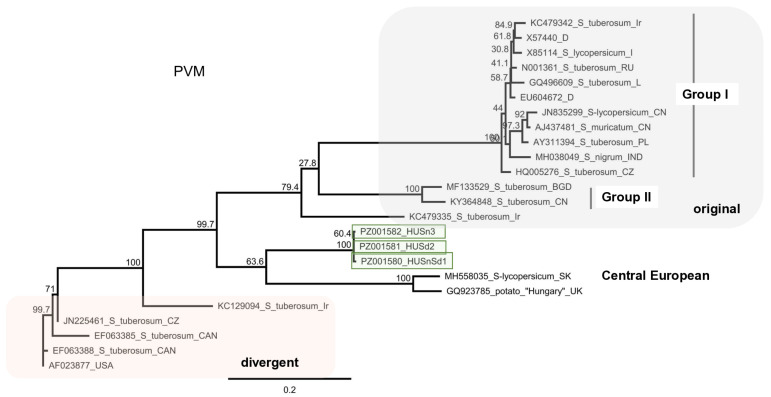
Phylogenetic analysis of the PVM variants (PZ01161-63). The analysis was conducted using Geneious Tree Builder with the Tamura-Nei model, the Neighbour-Joining method, and 1000 bootstrap replicates. The green box highlights the HUSn and Sd variants. The sequences are indicated by their GenBank accession numbers, the name of the host plant species, and their geographical origin, with an abbreviation of the country of origin. Iran—Ir, Canada—CAN, Czech Republic—CZ, Poland—PL, China—CN, Latvia—L, Germany—D, Russia—RU, Italy—I, India—IND, Slovakia—SK, and Bangladesh—BGD.

Sequencing of the amplified PVM CP fragment showed that it is only 78% identical to the reference genome (Table S4). It showed the highest identity (81%) to the variants sequenced from tomato in Slovakia [50], and to a variant sequenced at FERA from potato as a “Hungarian” strain (Table S10). This clade seems to be divergent both from the original and the previously described divergent clade [63], as well as for the Group I, Group II [50], and they share their Central European origin (Figure 15).

#### 3.2.7. PVH

**Figure 16:**
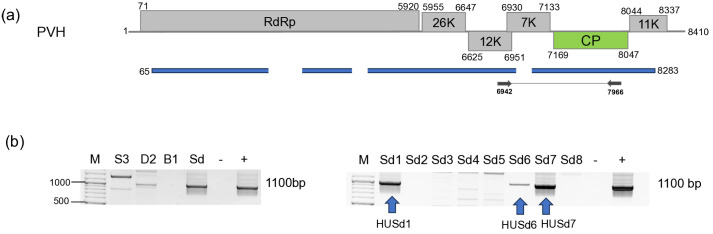
Summarised result of the HTS and its validation for the presence of PVH. (**a**) Schematic representation of the PVH genome and the positions of the primers used for RT-PCR validation. The dark blue line indicates the region covered by the contigs. (**b**) shows the result of the validation of the presence of PVH using RT-PCR. M represents the GeneRuler 100 bp Plus DNA Ladder; S3 indicates the *S. nigrum* pool of Field III; D2—*D. stramonium* pool of Field II; B1—*B. napus* pool; Sd—*S. dulcamara* pool; −/+ stands for negative and positive controls; Sd: 1–8 denote individuals of *S. dulcamara* at Field IV. The arrows show the PCR products, which were cloned and sequenced.

We identified 11 PVH contigs, which together with the 90,905 mapped reads covered the 90% of the PVH reference genome (Figure 16). RT-PCR validation identified three *S. dulcamara* plants infected with this virus.

**Figure 17:**
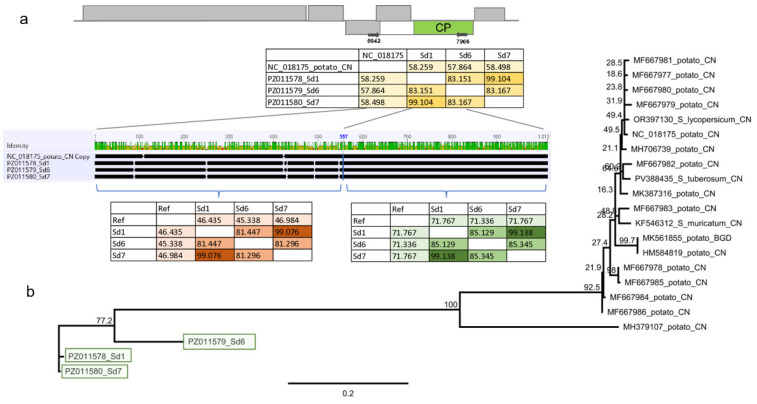
Sequence comparison and phylogenetic analysis of the PVH variants (PZ01158-60). (**a**) The cartoon represents the PVH genome, indicating the PCR amplified region. Pairwise sequence comparison of the cloned HU PVH variants with each other and with the reference genome is highlighted. The comparison was done for the full sequence (**upper** panel—yellow colour), the 5′ part (**left**, **lower** panel—red colour), or the 3′ part (**right**, **lower** panel—green colour). The intensity of the colour refers to the amount of identity. (**b**) Phylogenetic tree of the PVH variants. The analysis was conducted using Geneious Tree Builder with the Tamura-Nei model, the Neighbour-Joining method, and 1000 bootstrap replicates. The green box highlights the HUSd variant. The sequences are indicated by their GenBank accession numbers, the name of the host plant species, and their geographical origin, with an abbreviation of the country of origin. China—CN, Canada—CAN, and Bangladesh—BGD.

Cloning and sequencing the amplified PVH fragment showed that the three variants are different, showing 81–99% identity to each other (Table S11 and Figure 17).

The primers we designed amplified the 3′ part of the triple gene box and the 5′ half of the CP. Considering the full sequence of the amplified part, the Sd variants showed only 57–58% identity to the reference genome (Figure 17). The rules at the ICTV regulate the sequence of the CP when setting the species demarcation criteria for the virus species. When the CP coding part of the Sd variants was considered, they showed 71% identity to the reference genome (NC018175), which is at the border of the species demarcation criteria; however, this identity was only 45–46% when the 5′ half of the amplified part was compared. To investigate the phylogeny of the variants, we conducted the alignment of the CP coding sequence. The Sd variants are very different from the reference genome and share only 70–72% identity to the PVH variants sequenced so far, including the divergent PVH variants (MH379107). The Sd variants of the putative PVH clustered very distantly from the available PVH sequences, suggesting their independent origin (Figure 17).

#### 3.2.8. OxruMV1

**Figure 18:**
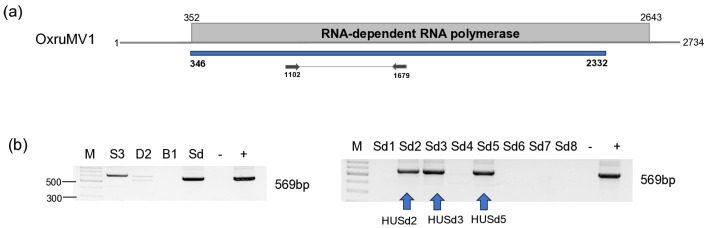
Summarised result of the HTS and its validation for the presence of OxruMV1. (**a**) Schematic representation of the OxruMV1 genome and the positions of the primers used for RT-PCR validation. The dark blue line indicates the region covered by the contigs; (**b**) shows the result of the validation of the presence of BBWV2 using RT-PCR. M represents the GeneRuler 100 bp Plus DNA Ladder; S3 indicates the *S. nigrum* pool; D2—*D. stramonium* pool; B1—*B. napus* pool from Field III; Sd—*S. dulcamara* pool from Field IV. −/+ stands for negative and positive controls; Sd 1–8 denote individuals of *S. dulcamara* at Field IV. The arrows show the PCR products, which were cloned and sequenced.

In our data, we identified six contigs and more than 400,000 reads mapped to the genome of OxruMV1, covering more than 72% of its genome. RT-PCR validated the presence of a mitovirus in *S. dulcamara*. Testing the individuals revealed that three of them were infected with this virus (Figure 18).

**Figure 19:**
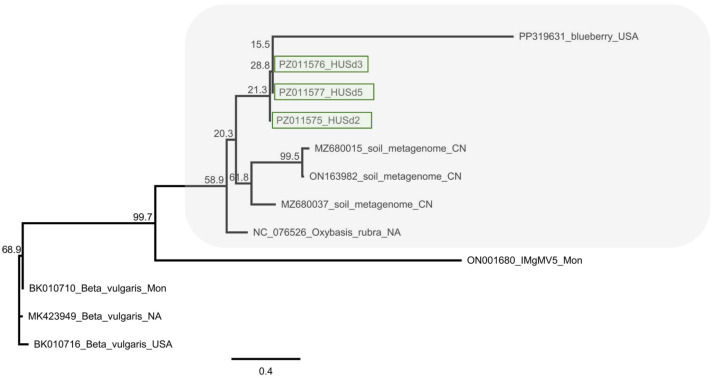
Phylogenetic analysis of the OxruMV1-like HUSd variants (PZ011575-77). The analysis was conducted using Geneious Tree Builder with the Tamura-Nei model, the Neighbour-Joining method, and 1000 bootstrap replicates. The green box highlights the HUSd variant. The sequences are indicated by their GenBank accession numbers, the name of the host plant species, and their geographical origin with an abbreviation of the country of origin: USA—United States of America, CN—China, Mon—Mongolia.

Sequencing the amplified part of the HUSD variants showed that they are not identical, but very similar (having more than 98% identity) (PZ011575-77) and showed the highest identity to the OxruMV1 reference genome (higher than 74%) (Table S12) (Figure 19).

The HUSD variants clustered in a clade, including a mitovirus identified in blueberry in the USA (Blueberry mitovirus 1 (PP319631)) and several mitoviruses, which were described during a soil metagenome study in China [56] and in Mongolia (Inner Mongolia grassland mitovirus 5 [64]). Among them, the only virus accepted by the ICTV that also had a reference genome was OxruMV1, with which the HUSd variants showed the greatest identity.

#### 3.2.9. TVCV

**Figure 20:**
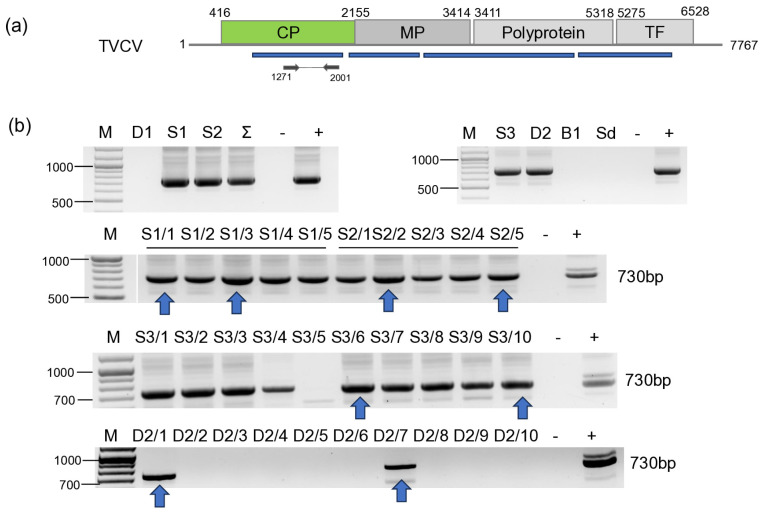
Summarised result of the HTS and its validation for the presence of TVCV. (**a**) Schematic representation of the TVCV genome and the positions of the primers used for RT-PCR validation. The dark blue line indicates the region covered by the contigs. (**b**) shows the result of the validation of the presence of LBVaV RNA1 and RNA2 using RT-PCR. M—represents the GeneRuler 100 bp Plus DNA Ladder; S3 indicates the *S. nigrum* pool; D2—*D. stramonium* pool; B1—*B. napus* pool from Field III; Sd—*S. dulcamara* pool from Field IV. −/+ stands for negative and positive controls; Sd/1–8 denotes individuals of *S. dulcamara* at Field IV. The arrow shows the products which were sequenced.

Validation of the presence of TVCV revealed its presence in all three tested *S. nigrum* populations and in two *D. stramonium* plants (Figure 20). Sequences of the PCR products amplified by TVCV-specific primers show a very high level (3.2–10.9%) of ambiguity (Table S13), suggesting that they could arise from different sources, integrated in the host genome.

Sequences of the pararetroviral sequences showed 79–97% identity to each other, while their identity to the reference genome was 67–82%. Interestingly, they showed slightly higher identity (68–83%) to the pararetroviral elements sequenced in *N. tabacum* [65]. These features indicate that TVCV sequences are integrated not only into the genome of cultivated plants, but also into the genome of wild solanaceous hosts.

## 4. Discussion

During our study, we investigated viromes of solanaceous plants using HTS. Although we sampled only 15 plants in 2022 and 21 plants in 2023, representing four species, we found the presence of nine viruses (Table 2).

We did not find any virus in the tested *B. napus* individuals. The infection rate of the solanaceous weeds was usually very low: one, two, three individuals out of the tested five, eight, ten were infected, except in the case of PVM and TVCV. All of the tested *S. nigrum* and *S. dulcamara* populations were infected in 2023 with PVM, and we found the presence of TVCV in all but one *S. nigrum*. HTS has proven its very high sensitivity as it detected the virus, even if only one individual in the pooled sample, out of the tested 15 or 31 was infected. Validation of the HTS using RT-PCR was successful in all cases, when we found at least one virus specific contigs with 0 E-value.

sRNA HTS, which we only proceeded with for the plants tested in 2022, indicated two different patterns. The strongest antiviral response was found in the case of CMV infection, when a high number of CMV-derived sRNAs, covering all three segments of the genome, mostly 21–22 nt long, were found, which is typical for RNAi found in the stage when the virus is actively replicating at the beginning of the infection cycle. In the case of BBWV1, the number of the virus-specific sRNAs is lower, but they are mostly 21–22 nt long, which indicates that it is still an active virus, but in a slightly later phase of the infection. This is the same pattern which we found when viromes of monocotyledonous plants were investigated [66]. There, virus-derived siRNAs of three viruses, including a luteovirus, barley virus G (BVG), showed two different patterns. In one case, the number of the virus-mapped siRNAs was very high, and their size was predominantly 21–22 nt long, while in the other case, the number of the virus-mapped siRNAs was low and their size was dominantly 24 nt long. This pattern was independent of the virus species and, according to our hypothesis, was more related to the stage of the virus infection. The low number and 24 nt long size of the TuYV-derived sRNAs could suggest that we found the later, persistent phase of the virus infection in this case. In the case of TVCV, we found a relatively high number of virus-derived sRNAs, which were mostly 24 nt long, specific for the activity of DICER3, which is specific for genome-integrated invasive elements. The uniform infection of the *S. nigrum* population and the high level of ambiguity of the sequenced TVCV-derived PCR products suggest that TVCV is integrated into several points of its genome, similarly to how it was found to be integrated into the genomes of different solanaceous crops [67].

BBWV1 is a widespread virus, able to infect a diverse range of plant species, but most of its report confide to serological tests. The phylogenetic relationship of the virus has been analysed in details revealing its unusually high variability [19]. In line with its wide host range, it has been detected in weeds, including *S. nigrum* [6]. In Slovenia, it has been found in mixed samples of weeds, which pools contained *S. nigrum* indeed [20]. Surprisingly, we have found only one plant out of 15 tested ones infected with BBWV1, suggesting that the source of the infection can be something else and not the *S. nigrum* population itself. Unfortunately, we did not know how high or low the infection rate was in France when the tested population contained 100 individuals. Based on the phylogenetic analysis, we found that the HUSn strain is a reassortant. This finding supports the hypothesis of Ma and colleagues, who, when analysing only HTS data of the pooled sample, suggested a frequent occurrence of reassortants of BBWV1 [6]. This event could have happened earlier, during the evolution of this strain, when a single plant or vector was coinfected with different strains of the virus, belonging to different clades. BBWV1 can cause serious problems; it has been identified as a causative agent of an outbreak in *Capsicum*, which resulted in almost 100% loss in crop yield [68]. Its presence in a weed population is a warning signal.

CMV has a wide host range, and it has also been described from solanaceous weeds, including *S. nigrum* and *D. stramonium*. Surprisingly, we found only one plant infected in 2022, and CMV was not present in the tested plants in 2023. The HuSn strain clustered together with symptomatic isolates in IA subgroup. This strain, if vectored from the weeds surrounding the crops, could be dangerous, and this is why control of *S. nigrum* in the vicinity of the vegetable fields and greenhouses seems to be crucial in order to prevent unwanted damage to plants and the fruits. Although infection with CMV could induce the development of symptoms in the crops, its presence can be latent in the weeds. One of the reasons for this could be that their secondary metabolites can alleviate the symptoms of virus infection, as it was found in the case of *D. stramonium*, where the extract of this plant could suppress the severity of CMV infection in chilli [69].

We have found TuYV both in *S. nigrum* and *D. stramonium*. While this virus has been described from *S. nigrum* in the Czech Republic [27], according to our knowledge, this is the first description of its presence in *D. stramonium*. Unfortunately, the sequence from the *S. nigrum* variant from the Czech Republic is not available, but the HUSDSSn strain showed the highest identity to the variant, which was sequenced in that study, originating from the same geographical location. We found infection with this virus only in 2022, and not in 2023. In 2023, oilseed rape was voluntarily grown in field III, and we also collected three individuals during our sampling at that time. Interestingly, we did not find TuYV infection at that time either in *B. napus* and *S. nigrum*. TuYV is exclusively vectored by aphids, and the infection rate of the plants depends on the vector activity [70], depending on the vegetative season and weather conditions, why the sampling time could be critical for virus detection. The number and pattern of the TuYV-derived sRNA read suggest the low intensity of the antiviral silencing, suggesting that in this case, the infection reached its persistent state. We could validate the presence of the virus in two plants out of 15 tested. In the RNAseq, we found a low number of virus-derived reads, which did not cover the entire genome, supporting the above hypothesis that the virus infection reached a persistent state, when a low concentration of the virus did not further induce a high activity of the antiviral RNA.

In our study, LBVaV was found to infect *S. dulcamara*, which is its first description from this host, adding a new solanaceous plant to the virus host range. The virus has been described previously in Hungary from lettuce [28]. The genetic diversity of the virus is not very wide; the identity of the sequenced genome is more than 96% for both RNA1 and RNA2. Comparative phylogenetic analyses of LBVaV isolates from Australia, Japan, and Europe have revealed distinct clades that underscore its geographical strain differentiation [30,31]. The strain sequenced in our study clustered together with the Australian strains, which we cannot explain, but it could be the result of the fact that we only prepared the tree based on a relatively short Sanger verified sequenced part of the viral genome. The strains sequenced from Hungary from lettuce (MF196227) clustered very close to our isolate, having 98% identity (295/301) when we compared their overlapping part. The presence of the virus in *S. dulcamara* is unexpected. The plants which we sampled were grown on the side of Lake Balaton, in a very moist soil. The virus is vectored by a soil-living fungus, *Olpidium brassicae*, which transmits it to the plant from the root of an infected plant to the root of a non-infected one [71]. In this situation, it is very unlikely that the infected plant could serve as a virus reservoir to any crop, but the presence of the LBVaV in Hungary alarms the possibility of its emerging infection in lettuce in the future.

We have found the presence of an ObPV-like tobamovirus in two *D. stramonium* and one *S. dulcamara* plants. We think the virus we found is the *Solanum dulcamara* yellow fleck virus (SDYFV) [8]. In 1983 and 1987, two tobamoviruses, suspected to be a distinct ToMV strain, were described in Hungary. One of them (ObPV) originated from pepper and could break the N-gene-based tobamovirus resistance in pepper [34], while the other one (SDYFV) could not [8]. SDYFV have been isolated from *S. dulcamara* and was found to be widely distributed in the *S. dulcamara* population in the floodplains of the Tisza River. The properties of the two viruses (SDYFV-ObPV) and SDYFV have been compared [48]. They did not react with the ToMV antisera, excluding the possibility that they are a variant of that virus, but cross-reacted with each other, leading to the conclusion that they indeed are the same virus. The SDYFV, used in that study, originated from the same source as its original description [8], but there is still no sequence available from the original SDYFV. In this study, the ObPV-like tobamovirus was found on *S. dulcamara*, and it clustered with ObPV. The *S. dulcamara* population in which we found this tobamovirus was grown at the edge of Lake Balaton, a similar habitat which was described for SDYFV, which is why we think that this virus is indeed is the originally described SDYFV. Our results seem to widen the host range of SDYFV, as it was also found to infect *D. stramonium*. However, only the entire sequencing of this tobamovirus could fully answer this question, which we plan to do in the future.

We have found the infection of PVM in the population of *S. nigrum* and *S. dulcamara*, which were tested in the same year. The homogeneous infection of the population suggests a very strong infection pressure, possibly originating from the infected aphid population in that year. The PVM present was grouped with a divergent PVM strain sequenced in Slovakia from tomato and with a strain which, although sequenced in the UK, was marked as “Hungarian”. The strain, sequenced in India from *S. nigrum*, grouped with the original group of PVM, suggesting that it is not the host that determines strain specificity. PVM was found to infect *S. dulcamara* both in the USA and in Hungary [13,37], but this identification was done by serological methods, and does not allow us to compare the strain in this weedy host phylogenetically. Very close clustering and high identity of the strains which were sequenced from two different species, suggesting that we found and sequenced the PMV population that is present in the natural flora in Hungary. Favourable conditions for the aphids could help their population to vector PVM from the naturally growing weeds to the crops, which could be latent, but could strengthen the symptoms of other crop-infecting viruses. This could happen to the tomato plants in Slovakia, where the T20 strain, clustering with the variants which we found, was present in a symptomatic tomato plant showing mild symptoms [50]. As there is no clear evidence on how harmful the presence of PVM is to the potato, its presence is not allowed in the propagation material. At Keszthely, where we collected our samples, there is a potato breeding station which also produces tubers for propagation. The presence of PVM in the weeds growing in this location is a warning signal. The distance between the tested *S. nigrum* population and the *S. dulcamara* population was more than 1 km, but we found very similar, almost identical strains of PVM in them, which suggests a common origin. The tuber production should be maintained in a crop rotation system to avoid the cumulative infection of pathogens in the propagation material, but the propagation field could be present in close vicinity to the natural habitats of these weeds, which means a constant infection risk, so the presence of the insects should be controlled.

PVH is a virus described in China more than a decade ago using traditional virus investigation methods [38]. Since then, even in the HTS area, it was only described from Asia, in potatoes, tomatoes, and pepino, and seems to be lacking from the other parts of the world. The PVH variant we sequenced is the first report from Europe, but because the identity of the HUSd variants is below the species demarcation criteria in the Carlavirus genus, and they were found in a different host: *S. dulcamara*, it is possible that we sequenced a new viral species, which is a very close relative of PVH.

In three *S. dulcamara* plants, we detected the presence of a mitovirus. Based on its identity, it can be a distinct variant of OxruMV1. Mitoviruses of plants were described less than a decade ago [43]. In parallel with the increased use of HTS and the investigation of metagenomes of plants and soil, more and more mitoviruses of the plant origin have been described. In this case, this mitovirus can also be part of the natural community of the tested *S. dulcamara* population. The virus was not present in all sampled plants, raising questions about its origin. Mitoviruses of fungi can alleviate their virulence when infecting their plant host, but we do not know yet how the presence of a mitovirus affects the physiology of its plant host, which is an interesting question to study in the future.

TVCV is a pararetrovirus, described to be from the Solanaceae family. Pararetroviruses can be episomal, replicating through an RNA intermediate, but can also integrate into the host genome. We sequenced RNAs, so when detecting TVCV-derived sequences, we could catch these forms. Investigating the RNAi response to endogenous plant pararetroviruses (EPRVs) Valii and colleagues found that during antiviral defence, most abundantly 22 nt long siRNAs are formed [72]. We found a remarkably different pattern, as the TVCV-derived siRNAs were mostly 24 nt long, indicating a host response typical for the genome-integrated elements. TVCV-like sequences were found to be integrated into several different places of the host genome as repetitive elements [65]. As the sequences of the PCR products amplified from single individuals contained a considerable level of ambiguities, we suspect that these TVCV elements are integrated into the host (*S. nigrum* and *D. stramonium*) genome. A recent study investigated the diversity of ERPVs in solanaceous crops, whereby tomato, pepper, eggplant and tobacco showed 88–100% in host identity, while the inter-species difference between the integrated TVCVs was 62–75%, and their identity to the TVCV reference ranged 69–80% [67]. We found that the identity of the TVCV sequences between the *S. nigrum* species was higher than 96, while the variants present in the two *S. dulcamara* differed by more than 20% (Table S13). While the investigated three *S. nigrum* populations were almost uniformly contained TVCV, it was present in only two *D. stramonium* individuals, raising the possibility that, indeed, a virus infection could happen. As there is no description about TVCV presenting in an episomal form in any host, we could have caught it in this situation, but we need further evidence to state that we found TCVC actively replicating in a weed. Investigating the effect of drought on the activation and copy number of ERPVs in tomato during different farming practices revealed that the copy number of the ERPVs changes only in the case of the sensitive genotype, suggesting that they could play a role in alleviating the effect of stress [67]. Detecting the presence of ERPVs in the genomes of solanaceous weeds suggests that these genome-integrated elements could shape their fitness under different stresses and help the weeds to survive during harsh conditions, but to test this hypothesis, further research would be needed in the future.

We found the presence of different viruses in the weeds growing in crop fields. If the viruses can infect the produced crop and the vectors of the virus are present, this could mean an infection risk and have consequences on the yield [73].

The plants sampled in the study grown at the close vicinity of the same geographical location showed markedly different virus profiles. The most infected species was *S. dulcamara*, and we found individuals that were coinfected with different viruses. This is not surprising, as it is a perennial plant, being able to collect and accumulate the infection of several different viruses during an extended period of time. The virome of the sampled species was uniform only in two cases. In the case of TVCV in *S. nigrum*, we think that the virus is integrated into the host genome, which explains this. Uniform infection rate was also found in the case of PVM, where a high infection rate of the aphid population infesting the plants could be the explanation. In the other cases, we found low, sporadic infection of the plants. The viruses found are not seed-transmitted, they are vectored by different organisms, like aphids and soil living *Ophidium* species, leading to a constant, but plasticly changing infection flow. In this initial study, we did not test the crops growing in the vicinity of the weeds, but the presence of this sporadic infection could happen not only between the weeds, but also between the weeds and the crops during one vegetation period. The climate is constantly shaped by global warming, and annual plants and vectors could more efficiently overwinter, and the virus reservoir effect of the weeds could extend to the next vegetation period. Alternative land use, like carbon farming, may promote weed and vector survival and extend their virus reservoir function, which warrants further investigation.

## 5. Conclusions and Dedication

Classical plant virologists have always been curious about the viruses present in our neighbourhoods. They used very sophisticated methods, including biotests on sensitive hosts and electron microscopy, and, later, produced by the virus-specific antibody, ELISA, to detect and classify the causal agents of diseases leading to loss of crop yields. Based on these techniques, they identified, characterised and taxonomically classified the most devastating viral agents. As curiosity never stops, they started to look not only at the crops for symptoms, but also at the weeds. Without sensitive and unbiased techniques, they could overlook the latent viruses, but they identified and described weed-infecting viruses. Some of these descriptions are only available in national periodicals, in different languages and are not digitised, which could lead to the loss of these very early and important discoveries. Knowledge of classical virologists is still a very important database of important results and deserves reinvestigation with the current techniques, which could lead to discoveries.

József Horváth, a classical virologist of Hungary, was born 90 years ago, and during his 46-year-long scientific career (1958–2004), he investigated the virus susceptibility of various plant species and infection of naturally growing solanaceous weeds. In his tremendous work, the susceptibility of 456 species of 66 genera belonging to 17 plant families, as well as 24 viruses belonging to nine virus groups was artificially tested, and the results were published in 18 papers [74]. He had fundamental results on the viromes of the solanaceous host in Hungary. One of his students, Pal Salamon, who passed away a year ago, was also a classical virologist who described the presence of several different viruses of solanaceous plants and weeds, but published most of his work in Hungarian. Reinvestigation of his observation led to the description of PrVI in *C. vitalba* [75], and we think that in this article, we present evidence that the SDYFV, originally described by him, is a different virus from ObPV.

To thank them for this invaluable knowledge, we would like to dedicate this research to them.

## Figures and Tables

**Table 1:** Characteristics, way of transmission, and occurrence of the viruses whose presence was identified in this study.

Name of the Virus	Acronym	Genus	Genome	Transmission	Weed (or Corp) spp. Infected	Occurrence
*Fabavirus alphaviciae*	BBWV-1	Fabavirus	bipartite, single-stranded, positive-sense RNA	aphids [18,19]	*S. nigrum*, *D stramonium*	France [6], Slovenia [20]
*Cucumovirus CMV*	CMV	Cucumovirus	tripartite positive-sense single-stranded RNA	over 70 aphid species	*S. nigrum*	Tunesia [4], India [5]
					*Solanum americanum*	Brazil [21] and China (GB MG014232)
					*Solanum ptycanthum*	Southern Illinois (USA) [22]
					*Solanum scabrum Miller*	Kenya [23]
					*D. stramonium*	Chile [24]
*Polerovirus TuYV*	TuYV	Polerovirus	single-stranded, positive-sense RNA	17 aphid species [25]	oilseed rape	Hungary [26]
					*S. nigrum*	Slovakia [17], Czech Republic [27]
*Varicosavirus lactucae*	LBVaV	Varicosavirus	bipartite, single-stranded, positive-sense RNA [28]	*Olpidium brassicae* [29]	lettuce	Hungary [28]
					*Sonchus oleraceus*	Spain [29,30,31]
					tomato	Slovakia [32]
*Tobamovirus obudae*	ObPV	Tobamovirus	single-stranded, positive-sense RNA	seed transmission [33]	pepper	Hungary [34]
					*S. dulcamara*	Hungary [8]
					*S.nigrum*	artificially, Hungary [35]
*Carlavirus misolani*	PVM	Carlavirus	single-stranded, positive-sense RNA	*Myzus persicae* [13]	potato	Hungary [36]
					*S. nigrum*	India [5]
					*S. dulcamara*	Hungary [37], New York State USA [13]
Potato virus H	PVH	Carlavirus	single-stranded, positive-sense RNA	not known	potato	China [38,39]
					tomato	China [40]
					peppino	China [41]
					potato	Bangladesh [42]
Oxybasis rubra mitovirus 1	OxruMV1	Mitovirus	single-stranded, positive-sense RNA	not known	*Oxybases rubra*	not specified [43]
*Solendovirus venanicotianae*	TVCV	Solendovirus	circular double-stranded DNA	not known	tomato, potato	genome integrated [44,45]

**Table 2:** Summary of the viromes survey of solanaceous weeds carried out in this study. Ratios indicate the number of the infected plants out of the number of the tested plants at the particular location. Green indicates the viruses which were first described in Hungary or from the tested host. Intensity of the infection is indicated by the shade of yellow. N/a means not applicable.

Virus Detected	2022			2023			
	Location I		Location II	Location III			Location IV
	*S. nigrum*	*D. stramonium*	*S. nigrum*	*S. nigrum*	*D. stramonium*	*B. napus*	*S. dulcamara*
BBWV1	0	0	1:5	n/a			
CMV	1:5	0	0				
**TuYV**	0	1:5	1:5				
**LBVaV**	n/a			0	0	0	1:8
**ObPV/SDYFV**				0	2:10	0	1:8
PVM				10:10	0	0	8:8
**PVH**				0	0	0	3:8
**OxruMV1**				0	0	0	3:8
**TVCV**	5:5	0	5:5	9:10	2:10	0	0

## Data Availability

Fastq files of the HTS are deposited into NCBI: SRA database (BioProject ID PRJNA1401399, BioSample ID SAMN54561009). Sanger sequences are available at NCBI GenBank (Accession numbers: PZ011567-PZ011584, and PZ051109-16).

## Supplementary Materials

The following supporting information can be downloaded at: https://www.mdpi.com/article/10.3390/v18040474/s1, Table S1: Initial statistics of the RNAseq and sRNA HTS; Table S2: Detailed results of the bioinformatic analysis of the RNAseq and sRNA HTS; Table S3: Sequences of the PCR primers used for virus detection with their appropriate references; Table S4: List of sequences which were deposited into the NCBI GenbBank; Table S5: Percent identity matrix of the BBWV1 sequences (a) RNA1 and (b) RNA2, which were considered in the phylogenetic analysis; Table S6: Percent identity matrix of the CMV sequences (a) RNA1, (b) RNA2 and (c) RNA3, which were considered in the phylogenetic analysis; Table S7: Percent identity matrix of the TuYV, which were considered in the phylogenetic analysis; Table S8: Percent identity matrix of the LBVaV (a) RNA1 and (b) RNA2, which were considered in the phylogenetic analysis; Table S9: Percent identity matrix of the viruses, which were considered in the phylogenetic analysis, when phylogeny of ObPV-like virus was investigated; Table S10: Percent identity matrix of the viruses, which were considered in the phylogenetic analysis, when phylogeny of PVM virus was investigated; Table S11: Percent identity matrix of the viruses, which were considered in the phylogenetic analysis, when phylogeny of PVH was investigated; Table S12: Percent identity matrix of the viruses, which were considered in the phylogenetic analysis, when phylogeny of an OxRuMV1-like virus was investigated; Table S13: Percent identity matrix of the TVCV sequences, which were considered in the sequence comparison.

## Abbreviations

The following abbreviations are used in this manuscript:

BBWV1	broad bean wilt virus 1
BLAST	Basic Local Alignment Search Tool
cDNA	copy DNA
CMV	cucumber mosaic virus
CP	coat protein
DCL	DICER like
EFSA	European Food Safety Authority
HeMV	Henbane mosaic virus
HTS	high-throughput sequencing
LBVaV	lettuce big-vein associated virus
LBVD	lettuce big-vein disease
LCP	long coat protein
MiLBVV	Mirafiori lettuce big-vein virus
MP	movement protein
MYFV	Melandrium yellow fleck
NCBI	National Centre fot Biotechnology Information
ObPV	Obuda pepper virus
ORF	open reading frame
OxruMV1	Oxybasis rubra mitovirus 1
PeMV	Pepino mosaic virus
PotLV	Pothos latent virus
PVA	potato virus A
PVH	potato virus H
PVM	potato virus M
PVX	potato virus X
PVY	potato virus Y
QC	quality control
RdRP	RNA dependent RNA polymerase
RNA	ribonucleic acid
RPM	reads per million
RT-PCR	reverse transcription-polymerase chain reaction
SCP	short coat protein
SoMV	sowbane mosaic virus
SRA	Sequence Read Archive
sRNA	small RNA
TMV	tobacco mosaic virus
TNA	Total nucleic acids
ToMV	tomato mosaic virus
TuYV	turnip yellows virus
TVCV	tobacco vein clearing virus

## Appendix A

**Table A1:** Basic information of the sampled plant species containing description of the symptoms, pooling strategy RNAseq and sRNA library codes.

Year	Field	Plant Species	Code of the Individual Plant	Symptoms	Pools	sRNA Library ID	RNAseq Library ID	Detected Viruses
**2022**	**I**	***Solanum nigrum***	S1/1	asymptomatic	**S1**	**KSOL**	**SOLKES1**	**TVCV**
			S1/2					**TVCV**
			S1/3					**TVCV**
			S1/4					**TVCV**
			S1/5					**CMV, TVCV**
		***Datura stramonium***	D1/1	asymptomatic	**D1**			
			D1/2					
			D1/3					
			D1/4					
			D1/5					**TuYV**
	**II**	***Solanum nigrum***	S2/1	asymptomatic	**S2**			**BBWV1, TVCV,**
			S2/2					**TVCV**
			S2/3					**TVCV**
			S2/4					**TVCV**
			S2/5					**TVCV, TuYV**
**2023**	**III**	***Solanum nigrum***	S3/1	Lcurl	**S3**	**n/a**	**SOLKES2**	**PVM, TVCV**
			S3/2	Ldef				**PVM, TVCV**
			S3/3	Lcurl, Ul				**PVM, TVCV**
			S3/4	Lcurl, Shi, Dw				**PVM, TVCV**
			S3/5	Dw, Lcurl, Ldef				**PVM**
			S3/6	Dw, Ac				**PVM, TVCV**
			S3/7	Ul, Dw, Lcurl				**PVM, TVCV**
			S3/8	Ul, Dw, Lcurl				**PVM, TVCV**
			S3/9	Dw, Ul, Lcurl, holes				**PVM, TVCV**
			S3/10	Dw, Ul, Lcurl, holes				**PVM, TVCV**
		***Datura stramonium***	D2/1	Ul	**D2**			**TVCV**
			D2/2	Dback				**SDYFV(ObPV)**
			D2/3	Fdef, Bspot, Ldef				
			D2/4	Lcurl, Ul, Chl				
			D2/5	Lcurl, Ul, Yflo				
			D2/6	Lcurl, holes, Ul				
			D2/7	Yflo				**TVCV**
			D2/8	Chl				
			D2/9	Ldef, Chl, holes				**SDYFV(ObPV)**
			D2/10	Ldef, Chl				
		***Brassica napus***	B1	No visible symtom	**B**			
			B2	No visible symtom				
			B3	holes				
	**IV**	***Solanum dulcamara***	Sd1	Chl, Ul, holes	**Sd**			**PVM, PVH**
			Sd2	Ul, holes, Ac				**PVM, OxruMV1**
			Sd3	Ldef, holes				**PVM, OxruMV1**
			Sd4	Bspots, Ldef, holes				**PVM, SDYFV(ObPV)**
			Sd5	holes, Nec				**PVM, OxruMV1**
			Sd6	Ldef, Lcurl, Nec, H				**LBVaV, PVM, PVH**
			Sd7	Ac, holes, Ldef				**PVM, PVH**
			Sd8	Ac, Nec				**PVM**

Abbreviations: Lcurl—leaf curl, Ldef—leaf deformation, Ui—uneven leaves, Shi—short internodes, Dw—dwarfism, Ac—anthocianin, holes—holes on the leaves, Dback—dying back, Fdef—flower deformation, Bspots—brown spots, Chl—chlorosis, Nec—necrosis.
